# Exposure to prenatal infection and the development of internalizing and externalizing problems in children: a longitudinal population-based study

**DOI:** 10.1111/jcpp.13923

**Published:** 2023-12-30

**Authors:** Anna Suleri, Anna-Sophie Rommel, Alexander Neumann, Mannan Luo, Manon Hillegers, Lotje de Witte, Veerle Bergink, Charlotte A. M. Cecil

**Affiliations:** 1 Department of Child and Adolescent Psychiatry/Psychology, Erasmus MC University Medical Center, Rotterdam, The Netherlands; 2 The Generation R Study Group, Erasmus MC University Medical Center, Rotterdam, The Netherlands; 3 Department of Psychiatry, Icahn School of Medicine at Mount Sinai, New York, NY, USA; 4 Department of Psychiatry, Erasmus MC University Medical Center, Rotterdam, The Netherlands; 5 Department of Epidemiology, Erasmus MC University Medical Center, Rotterdam, The Netherlands; 6 Department of Biomedical Data Sciences, Molecular Epidemiology, Leiden University Medical Center, Leiden, The Netherlands

**Keywords:** Maternal immune activation, neurodevelopment, maternal health, pregnancy, child health, psychopathology

## Abstract

**Background:**

A large body of work has reported a link between prenatal exposure to infection and increased psychiatric risk in offspring. However, studies to date have focused primarily on exposure to severe prenatal infections and/or individual psychiatric diagnoses in clinical samples, typically measured at single time points, and without accounting for important genetic and environmental confounders. In this study, we investigated whether exposure to common infections during pregnancy is prospectively associated with repeatedly assessed child psychiatric symptoms in a large population-based study.

**Methods:**

Our study was embedded in a prospective pregnancy cohort (Generation R; *n* = 3,598 mother–child dyads). We constructed a comprehensive prenatal infection score comprising common infections for each trimester of pregnancy. Child total, internalizing, and externalizing problems were assessed repeatedly using the parent-rated Child Behavioral Checklist (average age: 1.5, 3, 6, 10, and 14 years). Linear mixed-effects models were run adjusting for a range of confounders, including child polygenic scores for psychopathology, maternal chronic illness, birth complications, and infections during childhood. We also investigated trimester-specific effects and child sex as a potential moderator.

**Results:**

Prenatal exposure to infections was associated with higher child total, internalizing, and externalizing problems, showing temporally persistent effects, even after adjusting for important genetic and environmental confounders. We found no evidence that prenatal infections were associated with changes in child psychiatric symptoms over time. Moreover, in our trimester-specific analysis, we did not find evidence of significant timing effects of prenatal infection on child psychiatric symptoms. No interactions with child sex were identified.

**Conclusions:**

Our research adds to evidence that common prenatal infections may be a risk factor for psychiatric symptoms in children. We also extend previous findings by showing that these associations are present early on, and that rather than changing over time, they persist into adolescence. However, unmeasured confounding may still explain in part these associations. In the future, employing more advanced causal inference designs will be crucial to establishing the degree to which these effects are causal.

## Introduction

According to the developmental origins of health and disease (DOHaD) hypothesis, environmental exposures during sensitive periods of prenatal development may have short- and long-term consequences for an individual’s health. One environmental exposure that has gained increasing attention, especially in light of the recent COVID-19 pandemic, is that of infections during pregnancy (Fung et al., 2022; Han, Patel, Jones, Nielsen, et al., 2021). Experimental studies have shown that prenatal exposure to infections can impact various aspects of fetal development, including neurodevelopment, either directly or indirectly. When infectious agents cross the placental barrier, they can directly affect the developing fetal brain, leading to neurodevelopmental abnormalities. This group of infections is commonly referred to as TORCHES (Toxoplasmosis, Other [Syphilis, Varicella-zoster, Parvovirus B19], Rubella, Cytomegalovirus, Herpes Simplex) infections (al-Haddad et al., 2019; Han, Patel, Jones, & Dale, 2021). TORCHES pathogens can infect the placenta and gain access to the fetal circulation, causing damage to developing neural cells and tissues. On the contrary, some infections do not pass the placenta but can still influence fetal neurodevelopment indirectly through maternal immune activation (Han, Patel, Jones, & Dale, 2021). For example, during a maternal infection, the maternal immune system may release various cytokines and inflammatory molecules as a defense response, which can cross the placenta and reach the developing fetus. Both direct fetal infection and maternal immune activation can trigger cascades of inflammatory responses in the developing brain, disrupting important developmental processes such as neuronal migration, synaptogenesis, and myelination (Estes & McAllister, 2016).

A large number of studies have reported a link between prenatal infections and an increased risk of neurodevelopmental disorders such as autism spectrum disorder (ASD) (Antoun et al., 2021; Fung et al., 2022; Han, Patel, Jones, Nielsen, et al., 2021; Jiang et al., 2016) and attention deficit hyperactivity disorder (ADHD) (Antoun et al., 2021; Fung et al., 2022; Han, Patel, Jones, Nielsen, et al., 2021; Zhu, Jiang, & Sun, 2021), but also psychiatric conditions that typically have an onset later in life, such as schizophrenia (Fung et al., 2022; Khandaker, Zimbron, Lewis, & Jones, 2013), psychosis (Khandaker et al., 2013), and mood disorders (Khandaker et al., 2013). As noted by several reviews and meta-analyses (including the most recent review summarizing 42 studies on prenatal influenza infection and any neuropsychiatric condition; Fung et al., 2022), most studies to date have focused on exposure to severe infections – using linkage to registry or hospital data – and clinical phenotypes (e.g., diagnosis of a psychiatric disorder), typically measured at a single time point. Earlier work from our group also observed an association between infections during pregnancy and behavioral problems in adolescence within the general population (Suleri et al., 2023). However, it is unclear whether this association is present earlier in life and whether it changes over time, that is, if the effects of prenatal infection on child psychiatric symptoms are temporally persistent or transient across development. Studying associations with repeatedly assessed psychiatric symptoms could shed light on potential periods of susceptibility. To our knowledge, only one study recently addressed this gap by examining the association between prenatal infection (yes/no) in the first trimester and the development of psychiatric symptoms from mid-childhood (age 5 years) to adolescence (age 17 years) in 1,000 mother–child dyads from the general population in Western Australia (Patel et al., 2020). The authors found that infection exposure during the first trimester was associated with an increase in internalizing and externalizing problems in the child over time.

These findings suggest that in utero exposure to common infections is prospectively associated with the emergence of psychiatric symptoms during development. However, several important questions have yet to be addressed, specifically: (1) Is there a dose–response relationship between prenatal infection and child psychiatric symptoms? Answering this question requires information on multiple types of prenatal infections modeled as a continuous exposure, to understand whether there is a linear relationship or if this association exists only above a specific exposure threshold; (2) Are associations already apparent in the first years of life? Early childhood is an important age for developmental, cognitive, and behavioral milestones. Examining the association between prenatal infection and child behavior from early childhood will allow us to better understand at what age the effect manifests and to identify potential early mechanisms that may be used for intervention targets (Scharf, Scharf, & Stroustrup, 2016); (3) Is the relationship between prenatal infection and child psychiatric symptoms stable or temporally dynamic across development (i.e., is the putative association linear or non-linear)? Answering this question is important to better understand risk trajectories following exposure to prenatal infections. (4) Does the timing of infection matter? Major (neuro)developmental changes occur throughout pregnancy, which may be differentially affected by infection exposure, with potentially different downstream behavioral outcomes. Finally, (5) to what extent are associations between prenatal infection and behavioral problems confounded by other maternal or child-related factors? It is important to consider potential alternative explanations that could explain the association between prenatal infection and child psychiatric symptoms.

One key consideration is the potential joint influence of genetic vulnerability, which might underlie both susceptibility to infections and psychopathology in offspring, thereby contributing to the observed association (Shorter et al., 2022). As such, observed effects of prenatal infection on child mental health outcomes may be, at least in part, explained by genetic factors, rather than environmentally driven. Furthermore, mothers who experience more frequent infections during pregnancy may have other pre-existing chronic illnesses that could contribute to psychiatric risk in offspring (Han, Patel, Jones, & Dale, 2021; Mor & Cardenas, 2010). Other potential key confounders in the association between prenatal exposure to infections and child psychiatric symptoms may be birth complications (e.g., preterm birth and low birth weight) as well as infections occurring during childhood (Eaton, Mortensen, Thomsen, & Frydenberg, 2001; Han, Patel, Jones, & Dale, 2021; Kemp, 2014; Köhler-Forsberg et al., 2019; Romero et al., 2007). However, little is known about the extent to which these variables may explain observed associations between prenatal infections and child psychiatric symptoms.

To address these gaps in the literature, we investigated the longitudinal association between exposure to prenatal infection (repeatedly assessed in each trimester) and child psychiatric symptoms across development (repeatedly assessed at five time points across an average age range from 1.5 years to 14 years), using data from a large-scale population-based cohort (~3,600 mother–child dyads). We studied prenatal infections using a comprehensive score including different types of infection and examined whether associations vary by timing of infection during pregnancy. We adjusted for a wide range of potential confounders, including chronic maternal illness (ranging from maternal medication use to (pregnancy) inflammatory conditions), birth complications (ranging from placental vascular resistance to birth weight), child polygenic scores for psychopathology derived from genomic structural equation modeling (gSEM) (Grotzinger et al., 2019), and childhood infections. Lastly, we investigated the potential moderating effect of child sex.

## Methods

### Study selection and participants

This study was conducted within the Generation R Study, a population-based cohort that investigates the health of mothers and children from fetal stage onward (Jaddoe et al., 2007, 2008, 2012). Pregnant women living in Rotterdam, the Netherlands, were recruited for the study from April 2002 to January 2006, resulting in a sample of 9,778 mothers. These mothers and their children have been assessed multiple times over the years. The Medical Ethics Committee of the Erasmus Medical Centre approved all study procedures. Written informed consent was obtained from all parents. We obtained assent from the children. More information about the Generation R study can be found elsewhere (Kooijman et al., 2016).

To be included in our study, participants had to meet two criteria: information on (1) maternal infection for each trimester of pregnancy (mothers had to be enrolled in the first trimester) and (2) parent-reported child psychiatric symptoms outcomes for one of the five time points had to be available. One twin or sibling was excluded from each pair based on data availability, or randomly in case data availability was equal.

### Measures

#### Prenatal infections

**Figure 1:**
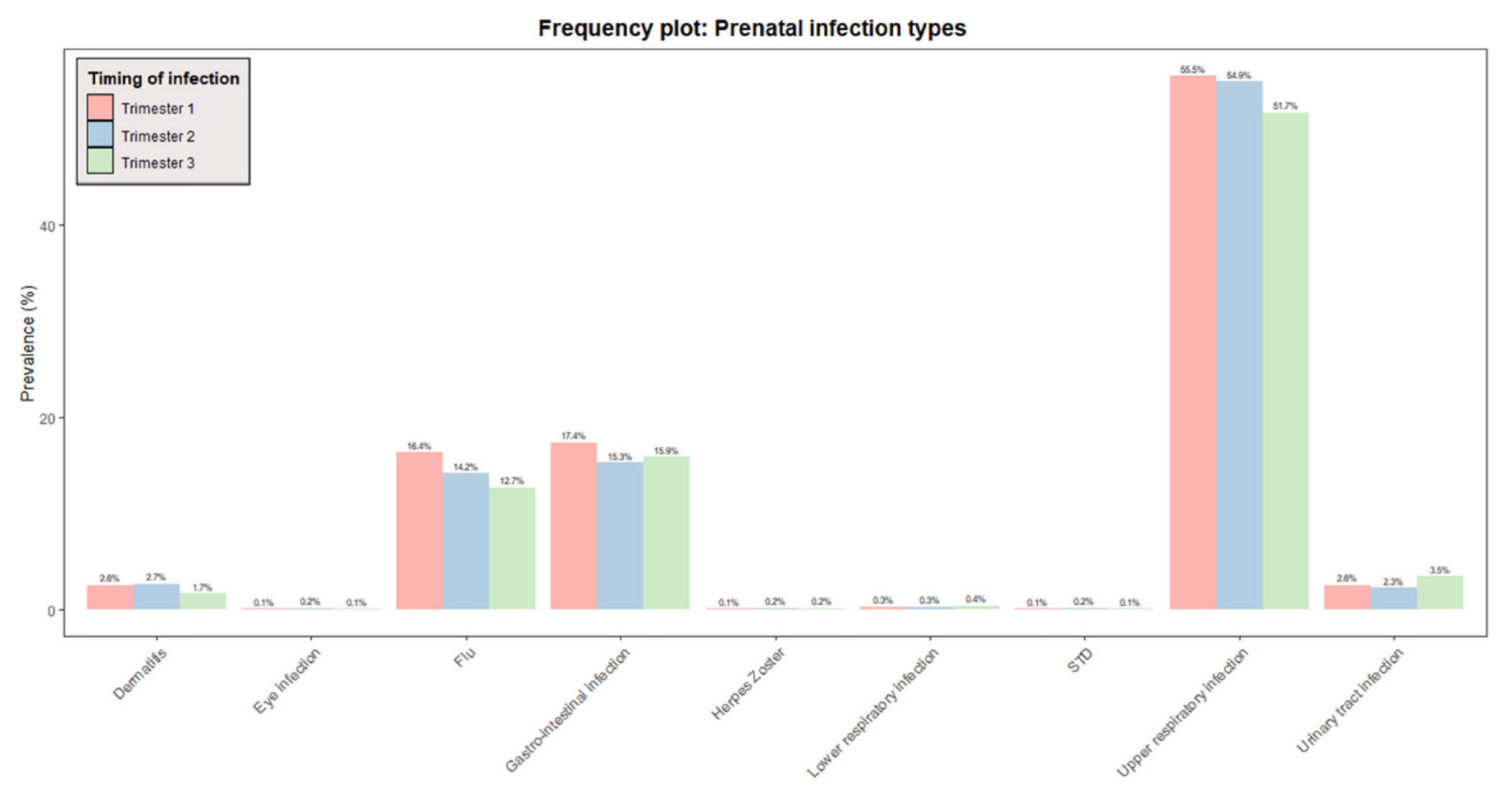
Distribution of infection types per trimester

To define our exposure, that is, prenatal infections, we used questionnaire data collected at three time points during pregnancy (one time per trimester). Women were asked to report on (1) any upper respiratory infections (pharyngitis, rhinitis, sinusitis, and ear infection), (2) lower respiratory infections (pneumonia and bronchitis), (3) gastrointestinal infections (diarrhea, enteritis), (4) cystitis/pyelitis, (5) dermatitis (boils, erysipelas), (6) eye infections, (7) herpes zoster, and (8) sexually transmitted diseases, and/or (9) flu, as well as (10) a period of fever (>38°C/100.4°F) within the past 2 (second trimester) or 3 months (first and third trimester). We created four sum scores: one for each trimester and one covering the entire pregnancy. Each reported instance of a condition within a specific trimester was given a score of one point in the trimester-based sum score. If a condition was not present, it was given a score of zero. Therefore, it was possible to get a total sum score of 30 points for the entire pregnancy for women with trimester-based sum scores available at all three time points (10 points per trimester). The distribution of each infection type per trimester can be found in Figure 1.

#### Child psychiatric symptoms

Child psychiatric symptoms were assessed using parent-reported ratings on the Child Behavioral Checklist (CBCL) (Achenbach & Rescorla, 2001). We administered the CBCL repeatedly from mean ages of 1.5 (= T1), 3 (= T2), 6 (= T3), 10 (= T4), and 14 (= T5) years using the CBCL version 1.5.-5 (T1–T3) and version 6–18 (T4, T5). The CBCL version 1.5–5 years consists of 99 items, and the CBCL version 6–18 years consists of 112 items. Both versions are reliable and valid questionnaires and thus, widely used for assessing common emotional and psychiatric symptoms (Achenbach & Rescorla, 2000, 2001). Psychometric studies that are based on empirical evidence have demonstrated that broad symptoms of mental health, and neurodevelopment can be grouped into internalizing and externalizing syndromes throughout childhood (Achenbach, 1991). Here, we used a sum score for the broadband scales: total psychiatric symptoms, internalizing (emotional) problems, and externalizing (behavioral) problems, in which higher scores indicate greater problems. We square-root transformed the outcomes to better satisfy normality assumptions of the residual distribution and random effects (Schielzeth et al., 2020). We further converted the scales of the behavior outcomes to Z-scores to standardize across different CBCL versions. Appendix S1 translates the definition of 1 standard deviation (*SD*) for each behavior outcome per time point. Figure S1 shows the time points of data collection for all covariates, as well as exposure and outcome variables.

#### Covariates

Child age was established from the date of birth and the date of questionnaire completion. Hospital registries provided information on child sex. Medical records were used to retrieve information on birthweight, gestational age at birth, placental weight at birth, 5-min Apgar score, caesarian delivery, premature ruptured membranes, pre-eclampsia, pregnancy-induced hypertension, gestational diabetes, and HELLP (hemolysis, elevated liver enzymes and low platelets) syndrome. Standardized fetal ultrasound examination was used to obtain two measures of placental vascular resistance, namely the pulsatility index in the arteria umbilicalis and resistance index in the arteria uterine (Gaillard, Arends, Steegers, Hofman, & Jaddoe, 2013). Placental growth factor was measured in non-fasting venous blood samples, and pH of the umbilical cord was measured in a cord blood sample in which gasses were checked with automated check machines (Gaillard et al., 2013).

Questionnaires at enrollment were used to assess maternal age, national background (‘Dutch’ or ‘non-Dutch’), household income (<€2,200/month or > €2,200/month), parental education (primary [no education or primary school], intermediate [secondary school or lower vocational training], and high [higher vocational training or university]), prenatal maternal psychoactive substance use (‘no’, ‘yes’), prenatal maternal psychoactive substance usage (‘no’, ‘yes, until pregnancy was known’, ‘yes, continued during pregnancy’), prenatal maternal tobacco use (‘no’, ‘yes, until pregnancy was known’, and ‘yes, continued during pregnancy’), and prenatal maternal alcohol consumption (‘none during pregnancy’, ‘drank until pregnancy was known’, ‘continued drinking occasionally’, and ‘continued drinking frequently’ [one or more glass/week for at least two trimesters]). Maternal psychopathology was further assessed at enrollment using the Brief Symptom Inventory (de Beurs, 2004). From this 53-item questionnaire (de Beurs, 2004), a Global Severity Index was calculated that served as a continuous score of prenatal maternal psychopathology with higher scores indicating more problems. Moreover, self-reported questionnaires at enrollment were used to collect information on maternal psychotropic medication use (includes selective serotonin reuptake inhibitors [SSRI], triptanes, antipsychotics, and tricyclic antidepressants [TCA]), maternal anti-infection or inflammation medication (includes non-steroidal anti-inflammatory drugs [NSAIDs], antibiotics, paracetamol, mucolytics, and antitussives), maternal corticosteroid use and inflammatory medical conditions (includes diabetes, arthritis, systemic lupus erythematosus [SLE] intestinal disorders, multiple sclerosis, and thyroid disorder). Mother-reported questionnaires were used to collect information on repeated measures of childhood infections (see Appendix S2 for more information).

Three cross-disorder polygenic scores (PGS) including neurodevelopmental, compulsive, and mood–psychotic PGSs were further used to account for child genetic confounding of psychiatric disorders, which were generated from our previous study (Hughes et al., 2023). In brief, gSEM was used to detect latent clustering of cross-disorder genomic data, based on genome-wide association studies of eight psychiatric disorders – ADHD, ASD, Tourette syndrome (TS), major depressive disorder (MDD), anorexia nervosa (AN), obsessive-compulsive disorder (OCD), schizophrenia (SCZ), and bipolar disorder (BIP). The gSEM identified three latent factors: (1) neurodevelopmental, which reflected loadings of ADHD, ASD, MDD, and TS PGSs; (2) compulsive, which reflected loadings of AN, OCD, and TS PGSs; and (3) mood–psychotic, which reflected loadings of BIP, MDD, and SCZ PGSs. We applied a liberal *p*-value threshold of 1.0 to include all SNP effects and to be consistent across the dimensions of psychopathology tested. Of note, PGSs based on this threshold have been shown to associate with the CBCL child behavior outcomes tested across multiple cohorts, including Generation R (Hughes et al., 2023). Detailed construction of the three gSEM-derived PGSs was described previously (Hughes et al., 2023). Maternal IQ (Raven’s Advanced Progressive Matrices Test set I [Raven, 1962]) was assessed when the child was 5–8 years old. Child IQ was assessed using the vocabulary, matrix reasoning, digit span, and coding subscales of the Wechsler Intelligence Scale for Children-Fifth Edition at age 13–16 years (Blok et al., 2022).

#### Statistical analyses

All analyses were performed using the R Statistical Software (version 4.1.2). We conducted a non-response analysis to explore potential selection bias. We tested the difference between the analytical sample and non-selected sample by using chi-squared test for categorical variables and two-sample unpaired *t*-test for continuous variables.

To investigate our primary research question, the longitudinal association between prenatal infection and child psychiatric symptoms, we applied linear mixed-effects models. We used the packages ‘*lme4’* to fit the models, ‘*broom*.*mixed’* for *p*-value computation, and ‘*ggplot2’* for visualization. As estimation method, we used restricted maximum likelihood (West, Welch, & Galecki, 2014). The exposure in our model was prenatal infection and each outcome (total problems, internalizing problems, and externalizing problems in the child) was analyzed in separate models. All models were adjusted for the following covariates: child sex, prenatal maternal age, maternal national background, maternal education, paternal education, household income, maternal IQ, child IQ, prenatal maternal psychopathology, and prenatal maternal tobacco, alcohol, and drug use. Additionally, we included a random intercept on the subject level to account for repeated measures of the outcome (child psychiatric symptoms). We further added a random slope for time to account for individual differences in the rate of change over time, which were not accounted for by the fixed effect predictors, meaning that we allowed the association between time and the outcome to vary randomly across individuals. Random intercept and slope were orthogonal to aid in model identification.

To better understand whether and to what extent there was a longitudinal association between prenatal infection and child psychiatric symptoms, we constructed one model focusing on the main effect of prenatal infection (see Appendix S3 for the equation) and one model including an interaction effect between prenatal infection and time (child’s age) (see Appendix S4 for the equation). We further explored in the main model (see Appendix S3 for the equation) whether there were non-linear effects of time by adding quadratic terms and cubic splines to see whether this improved the model fit. Hypothesis testing was conducted with the likelihood ratio test to establish the best-fitting model.

Subsequently, we ran a set of sensitivity analyses, where we repeated our main model (i.e., longitudinal effect of prenatal infection on child psychiatric symptoms over time [see Appendix S3 for the equation]) after additionally adjusting for (i) chronic maternal illness; (ii) birth complications; (iii) child genetic liability to psychopathology; and (iv) child exposure to infections during development. First, to adjust for chronic maternal illness, we included maternal psychotropic use (binary variable yes/no, based on any use of SSRI, triptanes, antipsychotics, or TCA), maternal anti-infection or inflammation medication use (binary variable yes/no, based on any use of NSAID, antibiotics, paracetamol, mucolytics, and antitussives), maternal corticosteroid use (binary variable yes/no), pregnancy inflammatory conditions (binary variable yes/no, based on preeclampsia, pregnancy induces hypertension, gestational diabetes, or HELLP syndrome), and inflammatory medical conditions (binary variable yes/no, based on diabetes, arthritis, SLE, intestinal disorders, multiple sclerosis, or thyroid disorder). Second, to adjust for birth complications, we included the following covariates in this model: placental growth factor, arteria umbilicalis pulsatility index, arteria uterine resistance index, 5-min Apgar score, umbilical cord blood pH, placental weight at birth, gestational age at birth and birth weight. Third, genetic liability to psychopathology was assessed using three gSEM-derived cross-disorder PGSs. Prior to analyses, PGSs were standardized (mean = 0, *SD* = 1) and residualized for the five first principal components to control for population stratification. Finally, to adjust for child infections, we included a sum score of infections occurring between birth and 9 years of age (see supplement Appendix S2 for more information on score).

To account for missing data in the covariates and outcomes, we applied multiple imputation using chained equations with the ‘*mice’* package for 30 datasets with 30 iterations. Missing values were estimated by generating multiple imputed datasets after which the results were pooled for all datasets using Rubin’s rules (Rubin, 2004). The maximum missingness was 75% in covariates and 33% in outcomes. To correct for multiple testing, the false discovery rate – Benjamini–Hochberg correction was applied for the main and sensitivity analyses (Benjamini & Hochberg, 1995). As such, p_FDR_ equal to or below 0.05 was considered as a significant result. The computer code used for this project is available at https://github.com/ajsuleri/Prenatal_infection_longitudinal_behavior.

## Results

### Demographic characteristics

**Figure 2:**
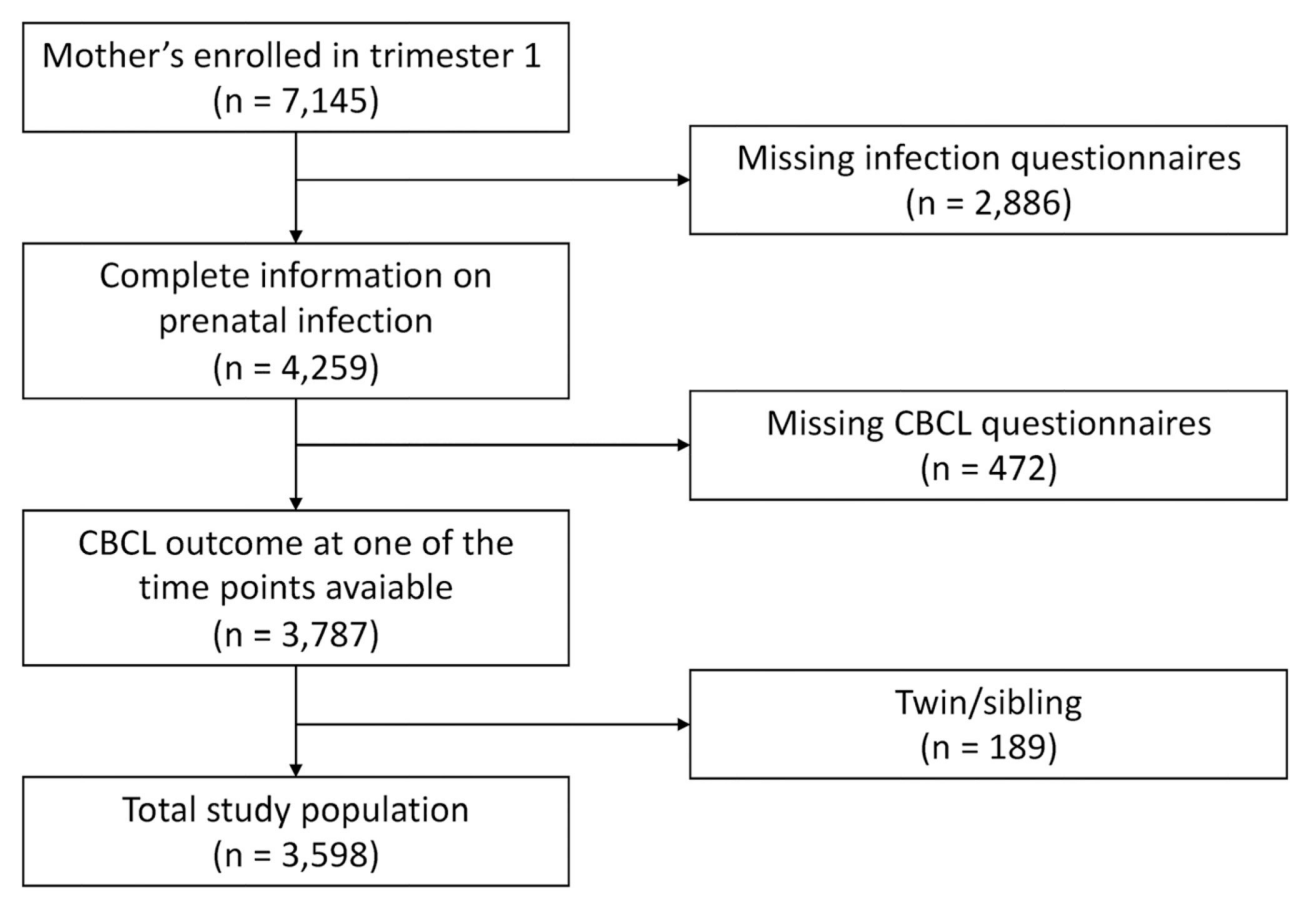
Flowchart study population

After applying the inclusion and exclusion criteria, the final sample size was 3,598 mother–child pairs (Figure 2). The baseline characteristics of the participants are represented in Table 1. Generally, included mothers were older (mean difference = 1.10 years, *df* = 8,835, *p* < .001), had a higher income (χ^2^ = 278, *df* = 1, *p* < .001), were more often of Dutch national background (χ^2^ = 409, *df* = 1, *p* < .001), had a higher education (χ^2^ = 389, *df* = 2, *p* < .001), consumed more alcohol during pregnancy (χ^2^ = 244, *df* = 3, *p* < .001), and had lower drug (χ^2^ = 7, *df* = 1, *p* = .01) and tobacco (χ^2^ = 32, *df* = 2, *p* < .001) use during pregnancy, compared with excluded mothers. The correlation between all variables can be found in Figure S2.

### Longitudinal associations between prenatal infections and child psychiatric symptoms

**Figure 3:**
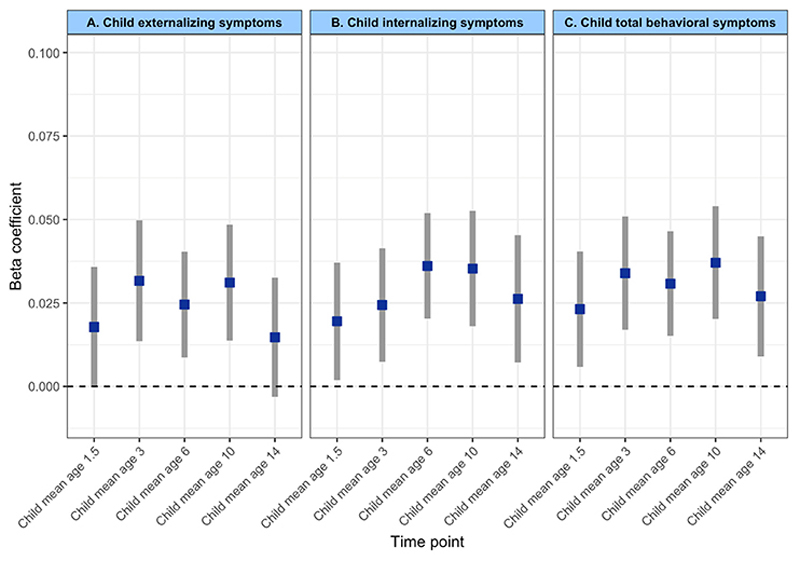
Association of prenatal infection on child psychiatric symptoms for each individual time point. For visualization purpose only the regression models were run for each time point individually (of note, as these are not mixed-effects models, these do not include a random intercept and random slope)

**Figure 4:**
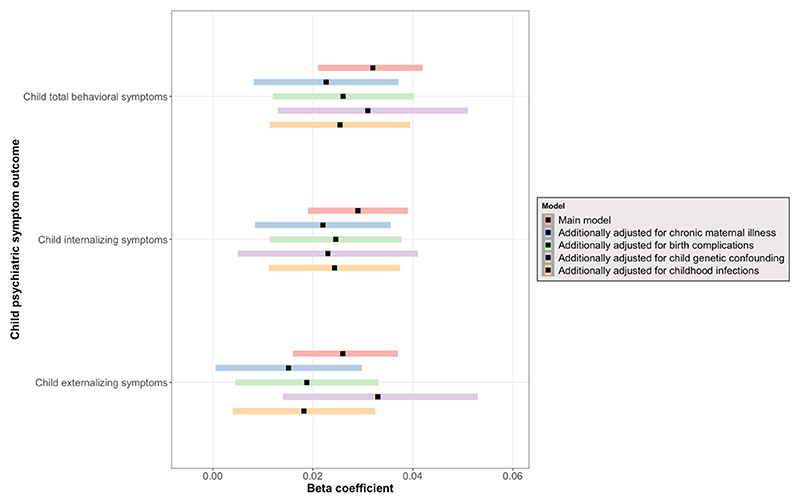
Forest plot showing effect estimates and 95% CI of main model and sensitivity analyses results

We observed a significant longitudinal association between prenatal infection exposure and child psychiatric symptoms (after adjusting for child sex, prenatal maternal age, maternal national back-ground, maternal education, paternal education, household income, maternal IQ, child IQ, maternal psychopathology, prenatal maternal tobacco, alcohol, and drug use, as well as repeated measures of outcome at subject level) (Table 2). Specifically, exposure to prenatal infection was positively associated with total psychiatric symptoms (*b* = .032, 95% CI 0.021–0.042, *p*_FDR_ < .001), internalizing problems (*b* = .029, 95% CI 0.019–0.039, *p*_FDR_ < .001), and externalizing problems (*b* = .026, 95% CI 0.016–0.037, *p*_FDR_ < .001) over time (Figure 3). There was no interaction between prenatal infection and time, meaning that prenatal infection was not associated with a change in child psychiatric symptoms over time (Table S1), but rather that the association remains temporally stable. For each outcome, the best-fitting model showed a linear trend over time. Adding a spline or quadratic term did not improve the model fit, meaning that a non-linear fit does not increase the explanatory power of the model. Moreover, we found that the longitudinal main effect of prenatal infection exposure on child psychiatric symptoms remained significant after additional adjustment for chronic maternal illness (Table S2), birth complications (Table S3), genetic liability for psychopathology (Table S4), and childhood infections (Table S5) (Figure 4 – Forest plot). We further observed no interaction between prenatal infection and child sex (Table S6) nor between prenatal infection, time, and child sex (Table S7), indicating that associations between prenatal infection exposure and child psychiatric symptoms over time are comparable between boys and girls.

### Trimester-specific effects

In our individual models (i.e., where trimester-based scores were modeled individually), we found that exposure to prenatal infection at any trimester was positively associated with higher total, internalizing, and externalizing problems over time (Table 3). In our mutually adjusted model (i.e., where trimester-based scores were modeled as multiple predictors to test the presence of trimester-specific effects, over and above other trimesters), we observed that prenatal infection in the first trimester was independently associated with child internalizing problems (*b* = .028, 95% CI 0.006–0.050, *p*_FDR_ = .021) (Table 4) over time. We further observed that prenatal exposure to infections in the second trimester was independently associated with child total psychiatric (*b* = .041, 95% CI 0.016–0.066, *p*_FDR_ = .003) and externalizing problems (*b* = .043, 95% CI 0.018–0.068, *p*_FDR_ = .002) (Table 4) over time. And lastly, we report that exposure to infections in the third trimester was independently associated with child total psychiatric (*b* = .033, 95% CI 0.009–0.057, *p*_FDR_ = .012) and internalizing problems (*b* = .039, 95% CI 0.016–0.062, *p*_FDR_ = .002) over time (Table 4). However, confidence intervals overlapped between different exposure trimesters and outcomes, which means that the effect differences are compatible with a true difference of zero (i.e., suggesting that effects between trimesters do not differ significantly from one another). A visual overview of all the results can be found in Figure S3.

## Discussion

In this large-scale population-based study, we examined whether exposure to prenatal infection prospectively associates with child psychiatric symptoms, measured repeatedly from toddlerhood to adolescence. In line with our hypotheses, we observed that exposure to prenatal infection was associated with higher total, internalizing, and externalizing problems across development, even after adjusting for a range of confounders including chronic maternal illness, birth complications, the child’s genetic liability to psychiatric conditions, and childhood infections. Prenatal infection was associated with stable and persistent child psychiatric symptoms over time, from early childhood to mid-adolescence (i.e., there was no change in the strength of associations over time). We further observed no interaction between prenatal infections, time, and child sex on child psychiatric symptoms. Moreover, when investigating trimester-specific effects, we showed that the effects of prenatal infection on child psychiatric symptoms are likely not significantly different between trimesters.

Our results show that exposure to infections in utero is associated with higher total, internalizing, and externalizing problems in the child over an average time span of 14 years, starting at the age of 1.5 years. This is consistent with findings from a recent longitudinal population-based pediatric study (Patel et al., 2020), which observed an increase in child psychiatric symptoms over an average time span of 12 years, starting at the age of 5 years, after prenatal exposure to infections. Interestingly, however, we did not identify an interaction between prenatal infection and time in our model, suggesting that prenatal infection exposure is not associated with an increase or decrease (i.e., change in slope) in child psychiatric symptoms over time (i.e., temporally invariant association) in this cohort. In other words, our findings indicate that the association between prenatal infection and child psychiatric symptoms manifests early in life and persists at least into adolescence.

It is noteworthy that, despite evidence that the neurodevelopmental PGS was strongly associated with child psychiatric symptoms within this sample in a previous study (Hughes et al., 2023), our findings remained consistent after adjusting for this score, as well as two other cross-disorder PGSs for psychiatric disorders. Yet, it should be noted that the PGS approach captures only a small fraction of genetic effects, and thereby does not fully account for potential genetic confounding (e.g., due to rare variants). Another important note is that not all psychopathology was measured (such as broad anxiety) in the PGS. However, we used gSEM-derived PGSs that index three major dimensions of genetic risk sharing across eight psychiatric disorders, which increases the accuracy of polygenic scores.

Our study yielded robust results after additionally adjusting for chronic maternal illness, birth complications, and childhood infections, which suggests that these confounding variables do not explain the association between prenatal exposure to infections and child psychiatric symptoms in our cohort. Moreover, we accounted for random variations between persons in our mixed-effects models. However, it is important to interpret these findings cautiously due to the low prevalence of birth complications and chronic maternal illness in our population-based cohort. We also did not have data on all relevant domains. For example, we had information on childhood infections but not on chronic child illnesses. Hence, our findings may still be attributed to other sources of unmeasured confounding, such as other genetic liabilities or health conditions (Lydholm et al., 2019). Furthermore, it is not possible based on our analyses to establish the causality of observed associations. In the future, the application of advanced causal inference methods, such as mendelian randomization, could provide valuable insights in this field.

Furthermore, in contrast to the other longitudinal study (Patel et al., 2020) that showed sex-specific effects for the association between infection count and child psychiatric symptoms, we observed no sex-specific effects in our study. It is unclear what explains this difference as the studies are similar in many ways. Namely, both studies included an equal proportion of boys and girls, were conducted in the general population, and used the CBCL to measure child psychiatric symptoms. Potential explanations may be differences in choices of covariates, as we additionally adjusted for socioeconomic status, maternal lifestyle factors, maternal psychopathology and cognition and child cognition in our main model, and chronic maternal illness, birth complications, genetic liability of the child for psychopathology, and childhood infections. Moreover, while the ages examined were partially overlapping between the two studies (child mean age 1.5–14 years vs. child mean age 5–17 years), the study by Patel et al. (2020) had a longer term follow-up (to age 17 years), which coincides developmentally with the increase in prevalence of certain psychiatric conditions (e.g., major depressive disorder) (Solmi et al., 2022). It will be of interest in the future to extend our analyses to a later age, as new time points become available in our cohort, to examine whether findings converge more over this time window.

An important question that arises from our findings is why exposure to infection prenatally has stable effects on child psychiatric symptoms over time. Partly, this could reflect the timing of the exposure. The prenatal period is critical for fetal brain development, particularly in the context of psychiatric disorders (Han, Patel, Jones, & Dale, 2021). Events occurring during this period may trigger biological adaptations (e.g., via epigenetic changes), leading to stable alterations in gene expression and downstream phenotypes, conferring long-term risk (Richetto et al., 2017). There are several potential mechanisms through which prenatal exposure to infections may influence fetal development (Han, Patel, Jones, & Dale, 2021). For instance, an infectious stimulus may directly affect the fetal central nervous system by crossing the placental barrier (i.e., vertical transmission). Additionally, maternal immune activation in response to prenatal infections may lead to an increase in both maternal and fetal immune cells, potentially disrupting neural circuit formation and other neurodevelopmental processes. The exact processes are unclear and may be dependent on the type of pathogen exposure. It is important to note, however, that these potential explanations assume a degree of causality which we are not able to establish in our study. Further investigation using comprehensive analysis of biological markers for both infectious pathogens as well as inflammatory markers such as cytokines will help to clarify the mechanisms underlying the observed relationship between prenatal infection and child development, through either vertical transmission of infectious pathogens or maternal immune activation.

In our individual models including trimester-based infection sum scores, we observed a similar association for each trimester on all child psychiatric symptoms outcomes. To establish whether these associations were independent (i.e., over and above the other trimesters), we also performed an analysis mutually correcting for all trimester-based infection sum scores. We found that the association between prenatal infection and child psychiatric symptoms is present at each trimester of pregnancy, although subtle differences in the magnitude of effects may exist. Future research is needed to replicate our findings and validate the robustness of our results concerning trimester-specific effects, in which infections could be subdivided by severity (acute or chronic infection across pregnancy) and type of infection. Due to the low prevalence of individual type of infections in our cohort, we were underpowered to examine the extent to which associations may vary by infection type.

### Strengths and limitations

The strengths of our study include the large prospective population-based cohort (*N* ~ 3,600) and the investigation of a broad range of infections at each trimester of pregnancy. Moreover, we used longitudinal modeling including psychiatric symptoms measured at five time points in which we corrected for relevant confounders including genetic liability to psychopathology, and multiple testing to minimize the probability of false positives. Our study also has several limitations. First, prenatal infections were recorded retrospectively via maternal self-report raising the possibility of recall bias. Yet, questionnaires were administered at relatively short intervals at the end of each trimester (~2–3 month recall) (McCarthy et al., 2022). Second, we were unable to investigate whether reported infections elicited a systemic immune response via biological markers. These, however, also have caveats including short half-life (e.g., CRP indexing acute inflammation; half-life of 9–12 h (Ablij & Meinders, 2002)) and healthy volunteer bias (ill participants will likely not visit the research center). Third, while we adjusted for child IQ, this measure was only available at the last time point (T5). Nonetheless, IQ is relatively stable over time (Deary, Pattie, & Starr, 2013; Franić et al., 2014). Fourth, some demographic measures differed between the participants who were included and excluded, which may limit the generalizability of our findings. Yet, we imputed our covariates and outcome to maximize our study population and to address selection bias. Lastly, while a longitudinal design helps to better characterize associations over time adjusting for confounding, it is still observational. Thus, we are not able to establish causality of the identified associations based on this study.

## Conclusion

In conclusion, our study adds to the evidence that not only severe infections but also mild infections during pregnancy might have an adverse impact on child neurodevelopmental outcomes. Moreover, our results imply that the associations between prenatal infection and child psychiatric symptoms are present in early toddlerhood and remain stable into adolescence (i.e., temporally invariant over an average period of 1.5–14 years). These stable associations remained after accounting for a range of potential genetic and environmental confounders. Additionally, we found that infections during all trimesters were associated with child psychiatric symptoms.

In the future, it will be important to validate these findings using more advanced inference approaches to establish causality. Bearing this proviso in mind, the identified associations raise several potential implications. Infections, especially upper respiratory infections, are extremely common during pregnancy and most pregnant women will experience these over the course of pregnancy. A stricter advice to avoid risk of infection to pregnant women might easily lead to unnecessary avoidance of social situations or unnecessary feelings of guilt after a common cold. Instead, global public health measures to generally lower infection risk for pregnant women might be a more fruitful approach. For example, due to the COVID-19 pandemic, it is an increasingly common practice in public spaces for people with respiratory infections to mask themselves and keep a distance, which might prove beneficial for pregnant women. Lastly, there is a large evidence base that general recommendations for a healthy lifestyle such as a balanced diet and exercise not only lower infection risk, but likely also have direct beneficial effect on the developing fetus. For example, our earlier work studying common infections during pregnancy and adolescent psychiatric problems observed that maternal psychopathology and lifestyle were among the factors that could attenuate the association between prenatal infection and psychiatric symptoms in adolescence (Suleri et al., 2023). Along with advice for the pregnant women, there may also be implications for clinical practice. Currently, in youth mental health care the focus lies on complications during delivery when collecting information on the developmental history of the child in the diagnostic process. Yet, it may also be helpful to supplement this with more information on the prenatal period, such as exposure to infections.

## Supplementary Material

Supplementary material

## Figures and Tables

**Table 1:** Baseline characteristics

	Study population *(n =* 3,598)
*General characteristics*	
Child’s sex, female (%)	1,792 (49.8)
Age mother at enrollment (mean, *SD) Birth characteristics*	30.6 (4.6)
Gestational age at birth	39.9 (1.6)
Birth weight (g) (mean, *SD*)	2,448.6 (548.8)
Caesarian delivery (yes, %)	427 (11.9)
Premature ruptured membranes (yes, %)	120 (3.3)
Placental weight at birth (g) (mean, *SD*)	636.9 (146.3)
Arteria umbilicalis (pulsatility index) (mean, *SD*)	1.2 (0.2)
Arteria uterine (resistance index) (mean, *SD*)	0.5 (0.1)
5-min Apgar score (mean, *SD*)	9.6 (0.7)
Umbilical cord pH (mean, *SD*)	7.3 (0.1)
Placental growth factor (pg/ml) (mean, *SD*)	54 (44.9)
*Maternal national background*	
Dutch (%)	2,276 (63.3)
Non-Dutch (%)	1,299 (36.1)
*Maternal education*	
Low (%)	186 (5.2)
Intermediate (%)	1,462 (40.6)
High (%)	1,913 (53.2)
*Maternal household income*	
<**€**2000 (%)	1,212 (33.7)
>**€**2000 (%)	2,203 (61.2)
*Maternal drug use*	
No (%)	3,337 (92.7)
Yes (%)	237 (6.6)
*Maternal smoking habits*	
Never smoked during pregnancy (%)	2,676 (74.4)
Smoked until pregnancy was known (%)	333 (9.3)
Continued smoking in pregnancy (%)	545 (15.1)
*Maternal alcohol consumption*	
Never drank in pregnancy (%)	1,378 (38.3)
Drank until pregnancy was known (%)	536 (14.9)
Continued drinking occasionally (%)	1,314 (36.5)
Continued drinking frequently (%)	347 (9.6)
*Maternal medical history*	
Psychotropic medication use (%)	62 (1.7)
Inflammation medication use (yes, %)	1,404 (39.0)
Corticosteroid medication use (yes, %)	203 (5.6)
Any pregnancy inflammatory condition (yes, %)	96 (2.7)
Any inflammatory medical condition (yes, %)	190 (5.3)
*Childhood infections*	
Childhood infections sum score (mean, *SD*)	3.1 (2.9)
*Exposure*	
Prenatal infection sum score spanning	3.0 (2.2)
pregnancy (mean, *SD*)	

**Table 2:** Longitudinal association between prenatal infection and child psychiatric symptoms

	β-coefficient	95% Confidence interval		*p*-Value	*p*_FDR_ Value
CBCL total psychiatric symptoms	.032	.021	.042	<.001*	<.001**
CBCL internalizing problems	.029	.019	.039	<.001*	<.001**
CBCL externalizing problems	.026	.016	.037	<.001*	<.001**

* *p* < .05.

** *p* < .05 after false discovery rate – Benjamini–Hochberg correction.

**Table 3:** Trimester-specific effects of prenatal infection

	β-coefficient	95% Confidence interval		*p*-Value	*p*_FDR_ Value
*Trimester 1*					
CBCL total psychiatric symptoms	.040	.018	.062	<.001*	<.001**
CBCL internalizing problems	.042	.021	.063	<.001*	<.001**
CBCL externalizing problems	.029	.006	.052	.012*	.022**
*Trimester 2*					
CBCL total psychiatric symptoms	.059	.037	.082	<.001*	<.001**
CBCL internalizing problems	.045	.023	.066	<.001*	<.001**
CBCL externalizing problems	.055	.032	.078	<.001*	0.001**
*Trimester 3*					
CBCL total psychiatric symptoms	.051	.028	.074	<.001*	<.001**
CBCL internalizing problems	.052	.030	.074	<.001*	<.001**
CBCL externalizing problems	.041	.019	.064	<.001*	<.001**

* *p* < .05.

** *p* < .05 after false discovery rate – Benjamini–Hochberg correction.

**Table 4:** Trimester-specific effects of infection (mutually corrected model)

	β-coefficient.	95% Confidence interval		*p*-Value	*p*_FDR_ Value
*Trimester 1*					
CBCL total psychiatric symptoms	.021	−.002	.044	.071	.110
CBCL internalizing problems	.028	.006	.050	.011*	.021**
CBCL externalizing problems	.011	−.013	.035	.359	.491
*Trimester 2*					
CBCL total psychiatric symptoms	.041	.016	.066	.001*	.003**
CBCL internalizing problems	.022	−.002	.045	.073	.110
CBCL externalizing problems	.043	.018	.068	.001*	.002**
*Trimester 3*					
CBCL total psychiatric symptoms	.033	.009	.057	.006*	.012**
CBCL internalizing problems	.039	.016	.062	.001*	.002**
CBCL externalizing problems	.025	.001	.048	.039*	.064

* *p* < .05.

** *p* < .05 after false discovery rate – Benjamini–Hochberg correction.

## Data Availability

Data from this study are available upon reasonable request to the director of the Generation R Study (generationr@erasmusmc.nl), subject to local, national, and European rules and regulations.

## Key points

- Existing studies and systematic reviews have reported associations between severe infections during pregnancy and child psychiatric conditions, typically by linking registry and hospital data at a single time point.
- In this large population-based study, we examine longitudinal associations between prenatal exposure to common infections and child psychiatric symptoms across development.
- Associations between prenatal infections and elevated psychiatric symptoms were already present in toddlerhood and persisted until adolescence.
- These associations remained after accounting for important confounders, including the child’s genetic liability to psychopathology, maternal chronic illness, birth complications, and infections during childhood.
- Prenatal infection may be an important risk factor for the emergence of child psychiatric symptoms from an early age.
