# Supplementary material for: Exposure to prenatal infection and the development of internalizing and externalizing problems in children: a longitudinal population-based study
Source: J Child Psychol Psychiatry. Author manuscript; Available in PMC 2024 Jul 1. (PMC7616076; doi:10.1111/jcpp.13923)
Supplement: Supplementary material — Appendix S1. Translating 1 SD to behavior scales. Appendix S2. Sum score for repeated measures of childhood infection. Appendix S3. Equation 1. Appendix S4. Equation 2. Table S1. Interaction between prenatal infection and time. Table S2. Longitudinal association between prenatal infection and child psychiatric symptoms (additionally adjusting for chronic maternal illness). Table S3. Longitudinal association between prenatal infection and child psychiatric symptoms (additionally adjusting for birth complications). Table S4. Longitudinal association between prenatal infection and child psychiatric symptoms after adjustment for genetic confounding. Table S5. Longitudinal association between prenatal infection and child psychiatric symptoms (additionally adjusting for child infections). Table S6. Interaction between prenatal infection and child sex. Table S7. Three-way interaction between prenatal infection, child sex, and time. Figure S1. Data collection time point for each study variable. Figure S2. Correlation matrix for prenatal infection, covariates, and child total psychiatric symptoms. Figure S3. Forest plot trimester-specific results. Panel A shows the models that were individually corrected, and panel B shows the results of the model that was mutually adjusted for all trimester infection sum scores. [file NIHMS1973420-supplement-Supplementary_material.docx]

**SUPPORTING INFORMATION**

**Appendix S1: Translating 1 SD to behavior scales**

The table below shows how much 1 standard deviation (SD) indicates for each behavior scale per time point.

| Outcome | Timepoint | 1 SD means … |
| --- | --- | --- |
| Total psychiatric symptoms | Age 1.5 | 14.8 |
|  | Age 3 | 13.7 |
|  | Age 6 | 16.4 |
|  | Age 10 | 15.5 |
|  | Age 14 | 16.3 |
| Internalizing problems | Age 1.5 | 4.3 |
|  | Age 3 | 4.2 |
|  | Age 6 | 5.8 |
|  | Age 10 | 4.8 |
|  | Age 14 | 5.8 |
| Externalizing problems | Age 1.5 | 6.5 |
|  | Age 3 | 6.1 |
|  | Age 6 | 6.6 |
|  | Age 10 | 4.9 |
|  | Age 14 | 5.2 |

**Appendix S2: Sum score for repeated measures of childhood infection**

A sum score for childhood infection was created based on information at eight different time points. In the table below, the time point, the question, the answer possibilities, and the scoring can be found. A total of 15 points could be scored, with a higher score representing a higher number of infections.

| **Timepoint** | **Question** | **Answer possibilities** | **Scoring** |
| --- | --- | --- | --- |
| 2 months | Has your child ever used one or more of the following medicines (antibiotics/penicillin)? | Never | 0 |
|  |  | Yes, for 1 period of sickness | 1 |
|  |  | Yes, for 2 or more periods of sickness | 2 |
| 6 months | Has your child ever used one or more of the following medicines (antibiotics/penicillin)? | Never | 0 |
|  |  | Yes, for 1 bout of illness | 1 |
|  |  | Yes, for 2 or more bouts of illness | 2 |
| 1 years | Has your child used one or more of the following medicines (antibiotics/penicillin) in the last 6 months? | Never | 0 |
|  |  | Yes, for 1-2 sickness periods | 1.5 |
|  |  | Yes, for 3-4 sickness periods | 3.5 |
|  |  | Yes, for more than 5 sickness periods | 5 |
| 2 years | Has your child been given one or more of the following medicines (antibiotics or penicillin) during the past year? | Never | 0 |
|  |  | Yes, 1-2 periods of illness | 1.5 |
|  |  | Yes, 3-4 periods of illness | 3.5 |
|  |  | Yes, 5 or more periods of illness | 5 |
| 3 years | Has your child been given one or more of the following medicines (antibiotics or penicillin) during the past year? | Never | 0 |
|  |  | Yes, 1-2 periods of illness | 1.5 |
|  |  | Yes, 3-4 periods of illness | 3.5 |
|  |  | Yes, 5 or more periods of illness | 5 |
| 4 years | Has your child been given one or more of the following medicines (antibiotics or penicillin) during the past year? | Never | 0 |
|  |  | Yes, 1-2 periods of sickness | 1.5 |
|  |  | Yes, 3-4 periods of sickness | 3.5 |
|  |  | Yes, 5 or more periods of sickness | 5 |
| 5 years | Was your child given antibiotics (for example, penicillin) during the past year because of a fever? | Never | 0 |
|  |  | Yes, 1-2 periods of illness | 1.5 |
|  |  | Yes, 3-4 periods of illness | 3.5 |
|  |  | Yes, 5 or more periods of illness | 5 |
| 9 years | In the past year did your child use any antibiotics (for example penicillin) for fever or infection? | Never | 0 |
|  |  | Yes, once or twice | 1.5 |
|  |  | Yes, 3 times or more | 3 |

**Appendix S3: Equation 1**

| Child_psychiatric_symptoms_j_ ~ child_age_ij_ + prenatal_infection_i_ + child_sex + maternal_age + maternal_national_background + maternal_education + paternal_education + household_income + maternal_IQ + child_IQ + maternal_psychopathology + maternal_prenatal_tobacco + maternal_prenatal_alcohol + maternal_prenatal_drug_use + (1 + child_age \|\| i) + $\varepsilon$_ij_ |
| --- |

**Equation 1**. Linear mixed-effects model. The *i* subscript indicates the subject, the *j* subscript indicates the timepoint. The term ‘Yij’ indicates the individual child psychiatric symptoms outcomes. The fixed effects part is the main part of the model that includes all the predictors. Here, the ‘prenatal_infection_i_’ is the term of interest for which the coefficient shows the average effect of prenatal infection on child psychiatric symptoms over time. In the random effects part of the model, the “1 + child_age || subject” term indicates the random intercept for subject (i.e., adjusting for the individual differences in the mean across all conditions; in the model intercept) and the random slope for subject (i.e., adjusting for the individual differences in the effect of the predictor).

**Appendix S4: Equation 2**

| Child_psychiatric_symptoms_j_ ~ child_age_ij_ * prenatal_infection_i_ + child_sex + maternal_age + maternal_national_background + maternal_education + paternal_education + household_income + maternal_IQ + child_IQ + maternal_psychopathology + maternal_prenatal_tobacco + maternal_prenatal_alcohol + maternal_prenatal_drug_use + (1 + child_age \|\| i) + $\varepsilon$_ij_ |
| --- |

**Equation 2**. Linear mixed-effects model. The *i* subscript indicates the subject, the *j* subscript indicates the timepoint. The term ‘Yij’ indicates the individual child psychiatric symptoms outcomes. The fixed effects part is the main part of the model that includes all the predictors. Here, the ‘prenatal_infection_i_ * child_age_ij_’ is the term of interest for which the coefficient shows the association between prenatal infection and change in child psychiatric symptoms over time. In the random effects part of the model, the “1 + child_age || subject” term indicates the random intercept for subject (i.e., adjusting for the individual differences in the mean across all conditions; in the model intercept) and the random slope for subject (i.e., adjusting for the individual differences in the effect of the predictor).

**Supplementary figures**

**Figure S1**. Data-collection time point for each study variable.


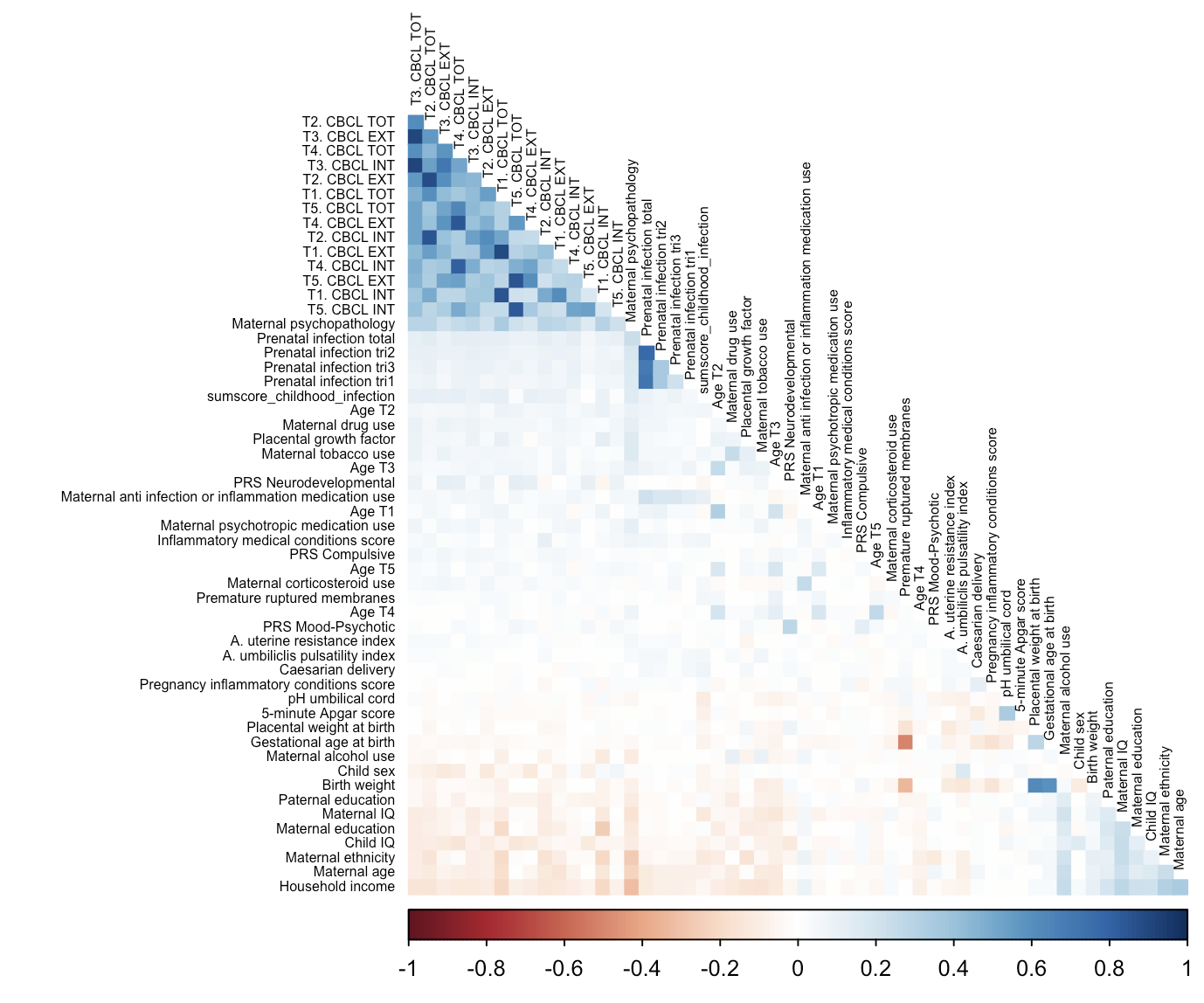


**Figure S2**. Correlation matrix for prenatal infection, covariates, and child total psychiatric symptoms.


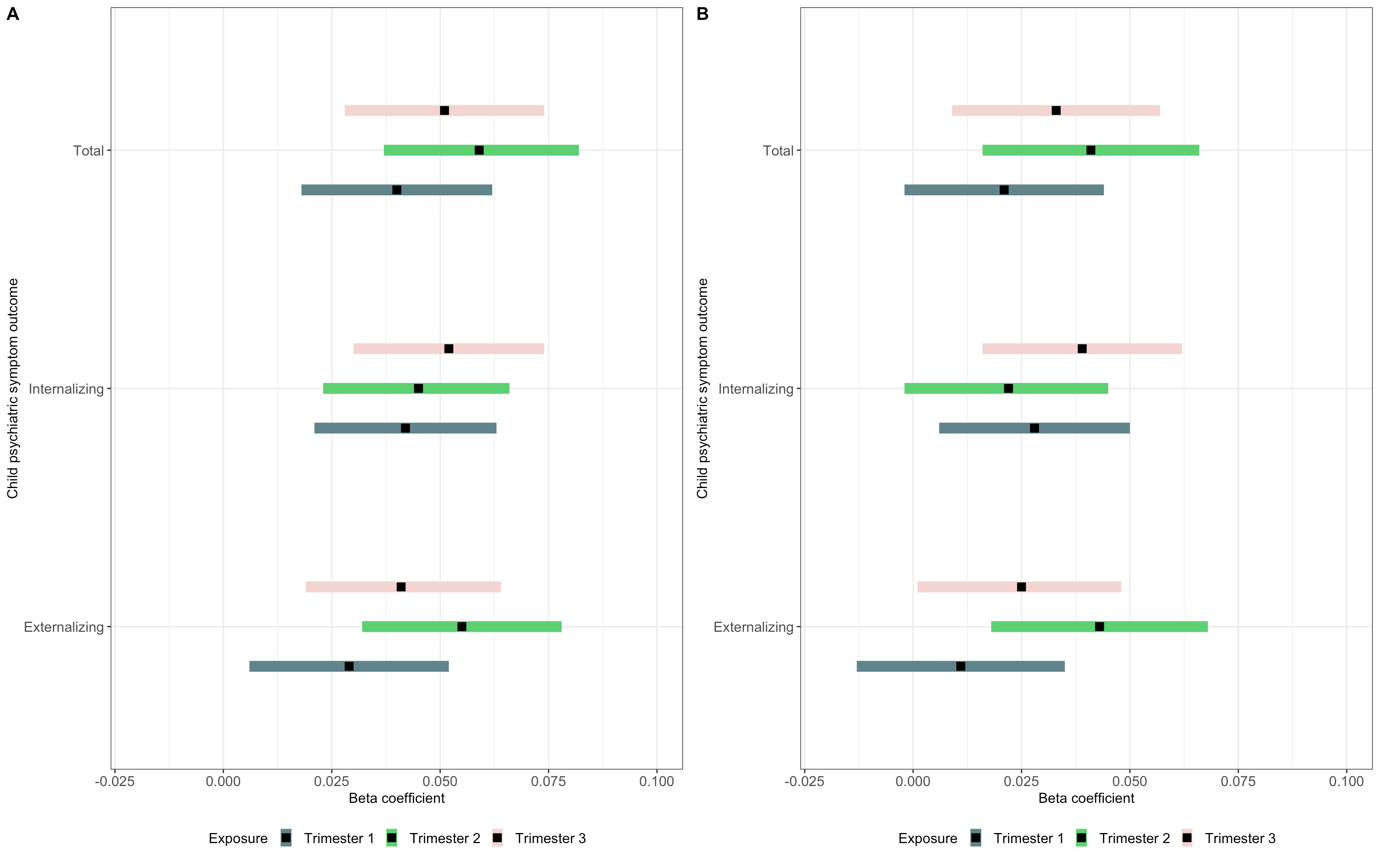


**Figure S3**. Forest plot trimester specific results. Panel A shows the models that were individually corrected, and panel B shows the results of the model that was mutually adjusted for all trimester infection sum scores.

**Supplementary tables**

**Table S1**. Interaction between prenatal infection and time

|  | $\boldsymbol{\beta}$-coefficient | 95% Confidence interval | | P-value | P_FDR_ value |
| --- | --- | --- | --- | --- | --- |
| CBCL total psychiatric symptoms | -.001 | -.002 | .001 | .553 | .691 |
| CBCL internalizing problems | .000 | -.002 | .002 | .818 | .944 |
| CBCL externalizing problems | -.001 | -.003 | .001 | .325 | .464 |
| *p < 0.05  **p < 0.05 after false discovery rate - benjamini-holchberg correction | | | | | |

**Table S2**. Longitudinal association between prenatal infection & child psychiatric symptoms (additionally adjusting for chronic maternal illness)

|  | $\boldsymbol{\beta}$-coefficient | 95% Confidence interval | | P-value | P_FDR_ value |
| --- | --- | --- | --- | --- | --- |
| CBCL total psychiatric symptoms | .023 | .008 | .037 | .002* | .003** |
| CBCL internalizing problems | .022 | .008 | .036 | .001* | .003** |
| CBCL externalizing problems | .015 | .001 | .030 | .042* | .042** |
| *p < 0.05  **p < 0.05 after false discovery rate - benjamini-holchberg correction | | | | | |

**Table S3**. Longitudinal association between prenatal infection & child psychiatric symptoms (additionally adjusting for birth complications)

|  | $\boldsymbol{\beta}$-coefficient | 95% Confidence interval | | P-value | P_FDR_ value |
| --- | --- | --- | --- | --- | --- |
| CBCL total psychiatric symptoms | .026 | .012 | .040 | <.001* | <.001** |
| CBCL internalizing problems | .025 | .011 | .038 | <.001* | <.001** |
| CBCL externalizing problems | .019 | .004 | .033 | .010* | .010** |
| *p < 0.05  **p < 0.05 after false discovery rate - benjamini-holchberg correction | | | | | |

**Table S4**. Longitudinal association between prenatal infection & child psychiatric symptoms after adjustment for genetic confounding

|  | $\boldsymbol{\beta}$-coefficient | 95% Confidence interval | | P-value | P_FDR_ value |
| --- | --- | --- | --- | --- | --- |
| CBCL total psychiatric symptoms | .031 | .013 | .051 | .001* | .002** |
| CBCL internalizing problems | .023 | .005 | .041 | .010* | .010** |
| CBCL externalizing problems | .033 | .014 | .053 | <.001* | .002** |
| ^†^Of note, the sample size for this sensitivity analysis was n=1,533  *p < 0.05  **p < 0.05 after false discovery rate - benjamini-holchberg correction | | | | | |

**Table S5**. Longitudinal association between prenatal infection & child psychiatric symptoms (additionally adjusting for child infections)

|  | $\boldsymbol{\beta}$-coefficient | 95% Confidence interval | | P-value | P_FDR_ value |
| --- | --- | --- | --- | --- | --- |
| CBCL total psychiatric symptoms | .025 | .011 | .039 | <.001* | <.001** |
| CBCL internalizing problems | .024 | .011 | .037 | <.001* | <.001** |
| CBCL externalizing problems | .018 | .004 | .032 | .012* | .012** |
| *p < 0.05  **p < 0.05 after false discovery rate - benjamini-holchberg correction | | | | | |

**Table S6**. Interaction between prenatal infection and child sex

|  | $\boldsymbol{\beta}$-coefficient | 95% Confidence interval | | P-value | P_FDR_ value |
| --- | --- | --- | --- | --- | --- |
| CBCL total psychiatric symptoms | -.001 | -.021 | .018 | .885 | .949 |
| CBCL internalizing problems | .002 | -.016 | .020 | .854 | .949 |
| CBCL externalizing problems | .001 | -.019 | .020 | .946 | .979 |
| *p < 0.05  **p < 0.05 after false discovery rate - benjamini-holchberg correction | | | | | |

**Table S7**. Three-way interaction between prenatal infection, child sex and time

|  | $\boldsymbol{\beta}$-coefficient | 95% Confidence interval | | P-value | P_FDR_ value |
| --- | --- | --- | --- | --- | --- |
| CBCL total psychiatric symptoms | .000 | -.003 | .003 | .997 | .998 |
| CBCL internalizing problems | -.001 | -.004 | .003 | .692 | .830 |
| CBCL externalizing problems | .001 | -.002 | .005 | .502 | .654 |
| *p < 0.05  **p < 0.05 after false discovery rate - benjamini-holchberg correction | | | | | |
